# 核糖核酸-蛋白质复合物规模化富集与鉴定技术的研究进展

**DOI:** 10.3724/SP.J.1123.2020.07019

**Published:** 2021-02-08

**Authors:** Zhiya FAN, Weijie QIN

**Affiliations:** 军事科学院军事医学研究院生命组学研究所,北京蛋白质组研究中心,国家蛋白质科学中心(北京),蛋白质组学国家重点实验室, 北京 102206; Beijing Institute of Lifeomics, Beijing Proteome Research Center, National Center for Protein Sciences(Beijing), State Key Laboratory of Proteomics, Beijing 102206, China; 军事科学院军事医学研究院生命组学研究所,北京蛋白质组研究中心,国家蛋白质科学中心(北京),蛋白质组学国家重点实验室, 北京 102206; Beijing Institute of Lifeomics, Beijing Proteome Research Center, National Center for Protein Sciences(Beijing), State Key Laboratory of Proteomics, Beijing 102206, China

**Keywords:** 核糖核酸结合蛋白, 紫外光交联和免疫沉淀, 规模化富集, 生物正交反应, 相分离, RNA-binding proteins, ultraviolet crosslinking and immunoprecipitation, large-scale enrichment, biorthogonal reaction, phase separation

## Abstract

核糖核酸(RNA)在细胞中并非单独存在,从它们产生到被降解的过程中与大量蛋白质发生相互作用,RNA结合蛋白(RNA-binding proteins, RBPs)能与RNA结合形成RNA-蛋白质复合物(RP复合物),并以这种复合物的形式发挥生理功能。RNAs或RBPs任一组分的异常与缺失都会影响RP复合物的正常生理功能,从而导致疾病的发生,如代谢异常、肌肉萎缩症、自身免疫性疾病和癌症。因此,定性定量分析RBPs及其在正常细胞和肿瘤细胞中与RNAs靶标之间的复杂相互作用网络有助于挖掘RP复合物在肿瘤发生发展中的作用,开发肿瘤生物标志物和新的治疗方式。要深入研究和理解RNAs与RBPs的相互作用网络,须依赖组学技术对RP复合物进行大规模鉴定。而作为在组学层面系统性解析RP复合物组成、含量和功能的第一步,大规模富集RP复合物极具挑战性。为了解决这一难题,研究者们发展了各种富集鉴定策略。该文针对RP复合物富集策略的最新进展进行了综述,包括紫外光交联和免疫沉淀(crosslinking and immunoprecipitation, CLIP)及其衍生技术、基于“点击化学”的富集策略和基于相分离的富集策略,比较分析了它们的技术原理、优缺点,以方便研究者们选择合适的策略来解决感兴趣的生物学问题。该文最后总结了当前的RP复合物富集方法仍然存在富集效率低和操作繁琐等亟需解决的技术挑战,为富集策略的发展提供了研究方向。

## Abstract (en)

Ribonucleic acid (RNA) rarely exists alone in the cell. RNAs interact with a variety of proteins and form RNA-protein complexes (RP-complexes) in every step of their life cycle, from transcription to degradation. These RP-complexes play key roles in regulating a variety of physiological processes. Defects in the composition and function of RP-complexes have been associated with many diseases, including metabolic disorders, muscular atrophy, autoimmune diseases, and cancer. It is hence evident that deciphering the highly complex interaction network of RNA-binding proteins (RBPs) and their RNA targets will provide a better understanding of disease development and lead to the discovery of new targets for cancer therapy. Large-scale identification of RP-complexes at the omics level is a prerequisite for obtaining insights into the complex RNA-protein interaction network. As the first step in omics-wide decoding of RP-complexes, enrichment and purification of RP-complexes is a highly challenging task. Recently, intensive efforts have been undertaken to better enrich and identify RP-complexes. Generally, the enrichment strategies can be classified into two major categories: in vitro and in vivo. Although it has been successfully applied in many studies, the in vitro transcribed bait RNA lacks modifications or structural similarity compared with its natural counterpart. Further, since the proteins relocate and remodel after cell lysis, the use of cell lysates as a protein source may result in capturing false interacting proteins that bind non-physiologically with the bait RNA. Finally, weak interactions between the non-covalently bound proteins and RNA require mild washing to remove non-specific binding, which needs careful optimization. However, substantial sample loss is inevitable. To overcome the disadvantages of in vitro approaches, in vivo cross-linking strategies that “freeze” natural RNA-protein complexes in intact cells via covalent cross-linking have become increasingly popular. The in vivo methods allow RNA to interact with proteins in the intracellular environment. Therefore, the RP-complexes formed under physiological conditions are more biologically relevant than those obtained by in vitro methods. We herein summarize recent in vivo methodological advances in the large-scale enrichment and identification of RP-complexes, including cross-linking and immunoprecipitation (CLIP) and related methods, click chemistry-assisted methods, and organic phase separations. CLIP involves irradiating living cells with 254-nm ultraviolet (UV) light to establish covalent bonds between RNA and proteins. This enables CLIP to purify RNAs bound to a specific RBP under conditions that are stringent enough to prevent co-purification of nonspecifically bound proteins or free RNAs. Since the original study, multiple variant protocols have been derived to increase both efficiency and convenience. Photoactivatable ribonucleoside-enhanced-CLIP (PAR-CLIP) introduces a variation in the crosslinking strategy. Cells were preincubated with photoactivatable ribonucleosides 4-thiouridine (4SU) or 6-thioguanosine (6SG), which enables protein-RNA crosslinking with 365-nm UV-A irradiation. It increases the efficiency of cross-linking between RNA and RBPs and is particularly valuable for studying the interactions between RBPs and nascent RNA. Using a click chemistry-assisted strategy, an alkyne modified uridine analog, 5-ethynyluridine (EU), was incorporated into nascent RNAs via metabolic incorporation in living cells. Combined with UV irradiation-based cross-linking, the alkyne-functionalized RNA and the bound proteins were purified in a poly A-independent fashion by the highly selective bioorthogonal copper (I)-catalyzed azide-alkyne cycloaddition using azide-modified beads. Thus, full lists of both coding and non-coding RNAs with their interacting proteins can be purified, which is a major methodological advance. Organic phase separation methods exploiting the physicochemical difference between cross-linked RP-complexes and free RNA and proteins do not require metabolic-based alkyne labeling or polyA-based RNA capture. Each method has unique strengths and drawbacks, which makes it important to select optimal approaches for the biological question being addressed. We hope that this review points out the current limitations and provides future directions to facilitate further development of methods for large-scale investigation of RP-complexes.

核糖核酸(RNA)是细胞基因组转录的产物,根据结构和功能的不同可分为编码蛋白质的信使RNA(mRNA)和非编码RNA(ncRNA), RNA参与很多重要的生命活动,是细胞中必不可少的一类生物大分子。RNA在细胞中并非单独存在,从它们产生到被降解的过程中与大量蛋白质发生相互作用,在真核细胞中存在上千种RNA结合蛋白(RNA-binding proteins, RBPs)与RNAs结合形成种类纷繁复杂的RNA-蛋白复合物(RP复合物),并以这种复合物的形式发挥生理功能。以mRNAs为例,pre-mRNAs被转录合成后经过5'端加帽、剪接、多聚腺苷酸化到成熟,再经过出核、定位和翻译到最终被降解,mRNAs的整个生命周期都依赖着多种mRBPs与之结合才能发挥作用^[1]^。同时,非编码RNA也在RBPs的参与下介导组蛋白修饰和基因调控过程^[2]^。

这些功能实现的前提是RP复合物的正确组装,RNAs或RBPs任一组分的异常与缺失都会影响RNAs的正常功能,从而影响基因表达^[3]^, RBPs还有可能通过干扰癌细胞能量代谢使癌症恶化^[4]^。这些都会导致生理过程紊乱和疾病的发生,包括代谢异常、肌肉萎缩症、神经系统疾病、自身免疫性疾病和癌症^[5,6,7]^。例如,RBP HuR(human antigen R)的过表达能在转录后水平调节信号通路,使癌细胞适应恶劣的肿瘤微环境,促进癌细胞增殖。在体外使用siHuR或小分子抑制剂选择性拮抗HuR或HuR-RNA相互作用能显著抑制肿瘤的生长。因此,定性定量分析RBPs的表达谱及其在正常细胞和癌细胞中与RNAs靶标之间的复杂相互作用网络有助于挖掘RP复合物在肿瘤发生发展中的作用,并为开发肿瘤生物标志物和治疗方式提供了新的思路。

目前研究者们已经不再满足于研究单个RP复合物的功能,在组学层面上研究和理解RNAs与RBPs的相互作用是必然趋势。生物质谱具有灵敏度高、动态范围宽、通量大的特点,是组学研究的必要分析手段。但由于RNAs与RBPs相互作用的动态性和网络复杂性,全面系统的阐述RP复合物的组成及动态变化并非易事。而作为系统性解析RP复合物组成、含量和功能的第一步,大规模富集RP复合物极具挑战性。为了解决这一难题,研究者们发展了各种富集鉴定策略,本文针对RP复合物富集策略的最新进展进行了综述,比较分析了它们的技术原理、优缺点及应用,并提出了需要解决的技术挑战,为富集策略的发展提供新的思路。

## 1 RP复合物富集策略

早期在富集RP复合物时通常利用RBPs与RNAs之间保持天然结合的特性在体外条件下实现富集,然而利用非内源性RNA和蛋白质,在非体内环境的结合会产生相当程度的假阳性结果,高洗涤强度也会导致RP复合物中结合力低的组分丢失。而体内条件下形成的RP复合物比通过体外方法获得的RP复合物更具有生物学相关性,能更真实地反映体内RNA-蛋白质相互作用的生理状态,近年最新发展的富集策略主要是在体内环境下实现RP复合物的富集。同样,为了克服因洗脱造成的部分RP复合物丢失的难题,需要增强核酸与蛋白质的相互作用。最简单有效的方法就是进行交联(cross-linking),主要分为化学交联和紫外光(UV)交联。化学交联通常采用甲醛试剂——一种双功能交联剂,可轻易渗透细胞并在0.2 nm以内的大分子之间形成可逆的共价键,因而会形成蛋白质-蛋白质复合物干扰RP复合物的鉴定。紫外光交联则是在RP复合物研究中应用更为广泛的“零距离”交联方式,UV可特异性地引发蛋白质与RNA之间形成不可逆的共价交联,从而排除在甲醛交联中不可避免的蛋白质-蛋白质交联,降低结果的假阳性。UV交联无疑成为体内研究RP复合物的基础,围绕UV交联诞生了许多经典的研究策略,下面将详细阐述。

### 1.1 UV交联和免疫沉淀及衍生技术

2003年Darnell等^[8]^提出了一种用于RP复合物富集的UV交联和免疫沉淀(crosslinking and immunoprecipitation, CLIP)策略,其目的是捕获并检测与特定蛋白质结合的RNA片段,他们使用CLIP策略联合Sanger测序鉴定到了340个与小鼠脑中剪接因子Nova1、Nova2相互作用的RNA序列。随后,Darnell团队又对细节进行了优化^[9]^, CLIP的技术路线是:首先通过UV(254 nm)照射使RBP与RNA共价交联,然后使用RNA酶(RNase)温和酶切,与RBP结合的RNA会因RBP的保护而留下一定长度的片段,再将RNA片段的3'-磷酸基团进行去磷酸化,防止RNA片段的环化自连接,而RNA片段的5'末端将进行放射性同位素标记(^32^P标记),接着用修饰有目标蛋白质抗体的微球/磁珠将目标RBP蛋白及RNA片段富集下来,再使用蛋白酶K将蛋白质降解,得到的RNA片段将采用逆转录-聚合酶链式反应技术(reverse transcription-polymerase chain reaction, RT-PCR)扩增,最后进行测序分析就可以得到目标RBP结合RNA的种类以及结合的位点信息,具体流程如图1。

**图1:**
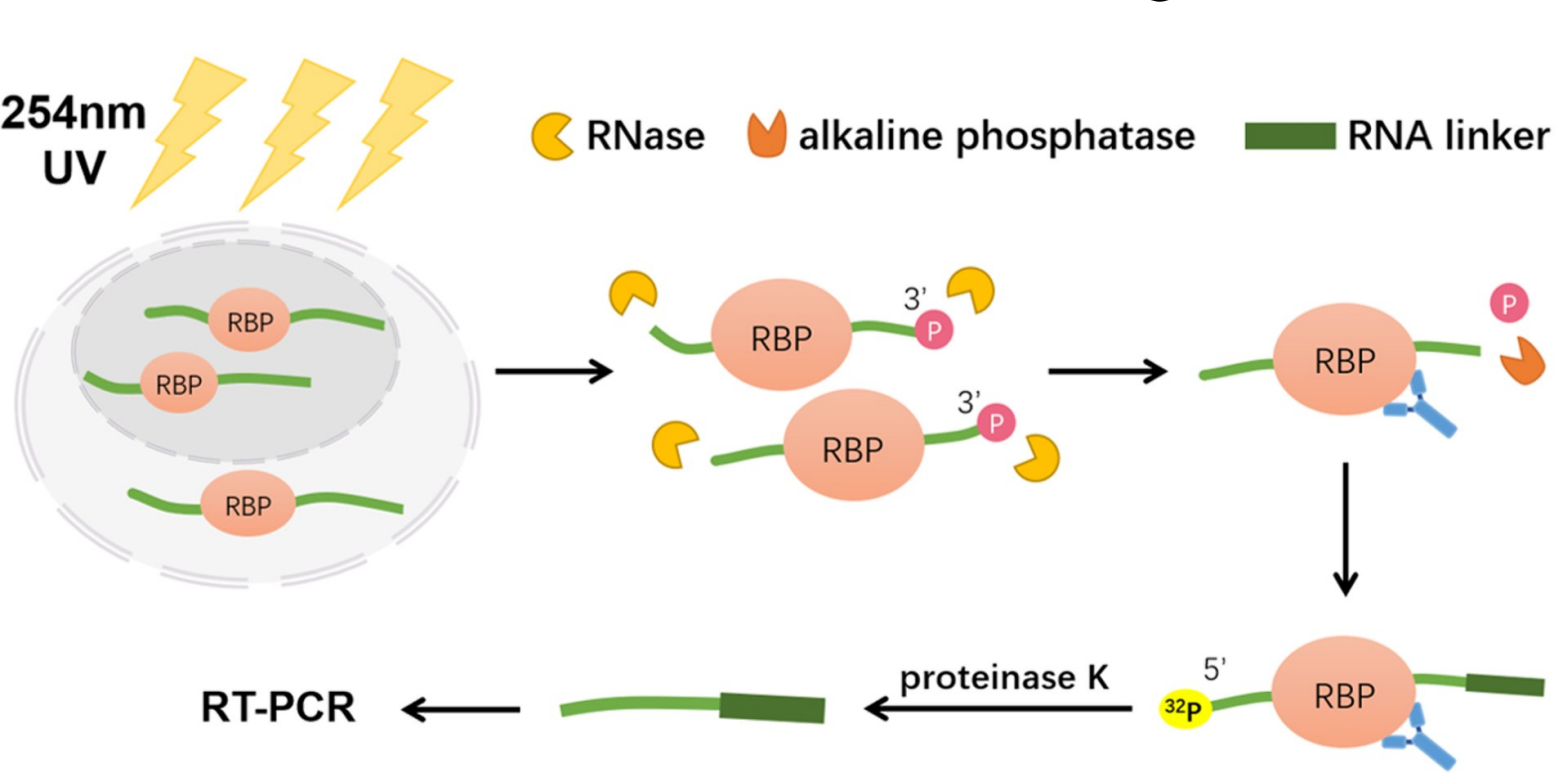
CLIP技术鉴定RNAs蛋白结合位点示意图

CLIP技术一经提出就获得了高度关注,但是该方法也面临着通量低、UV存在偏好性、穿透力弱、交联效率低(大约仅为1%~5%)等问题。尽管可以通过对组织样品低温研磨和不断混合使UV更高效地穿透细胞促进样品的均匀交联,但高能量UV长时间照射可能会导致RNA的降解^[10]^。在此基础上,越来越多的研究者投入研究并不断改进,产生了许多各具特色的衍生CLIP技术。

为了提高交联效率,Hafner等^[11]^发展了一种光活化核苷增强的CLIP策略(photoactivatable-ribonucleoside-enhanced crosslinking and immunoprecipitation, PAR-CLIP)。他们将光活性更强的核苷代谢进入RNA,可以使RNA和蛋白质在更长波长的UV(如365 nm)照射下交联。具有代表性的核苷有4-硫代尿苷(4-thiouridine, 4SU)和6-硫代鸟苷(6-thioguanosine, 6SG), 4SU比6SG的交联效率更高。与常规的254 nm UV交联相比,PAR-CLIP可将交联效率提高100到1000倍。此外,PAR-CLIP的另一项优势是,4SU与蛋白质发生交联后,该位点在逆转录时受到非交联寡核糖核苷酸背景的影响,多达70%的RNA序列中的尿嘧啶(uracil, U)被识别为胞嘧啶(cytosine, C),于是会得到相对应的cDNA序列中的胸腺嘧啶(thymine, T)到C的突变,由此可推测该位点是RBP的结合位点。但是PAR-CLIP技术也有一定局限性:由于需要在细胞培养时将4SU或6SG代谢进入RNA,此方法仅限于细胞水平,不适用于组织样品;细胞倾向于不使用非天然核苷酸类似物,这限制了4SU或6SG代谢进入细胞的效率;长时间的摄入4SU或6SG可能会引起细胞毒性^[12]^。因此,仍然需要新的方法来提高交联效率并且实现对RP复合物的更深覆盖。

CLIP及其衍生技术被广泛应用于酵母、真菌、哺乳动物的RNA-蛋白质相互作用研究中。值得一提的是,Castello等^[13,14]^利用UV交联结合oligo(dT)富集与质谱鉴定poly(A) RBP,发展了RIC(RNA-interactome capture)策略,可以大规模富集RBP。结合生物质谱技术,该策略在人宫颈癌细胞HeLa中鉴定到860个高置信的RBPs,极大地补充了人们对RBPs的认知。然而,这种方法基于RNA的poly(A)尾巴(主要是mRNA),而mRNA仅占细胞中RNA总质量的3%~5%^[3]^。并且不是所有mRNA都带有poly(A)尾巴^[15]^, poly(A)的长度也不尽相同^[16]^,导致部分mRNA也很难被oligo(dT)捕获。因此,RIC策略遗漏了大量RP复合物,无法鉴定ncRNA-蛋白质复合物乃至全类型RNA-蛋白质复合物。

### 1.2 基于“点击化学”的富集策略

鉴于RIC策略的局限性,最近一种基于代谢标记结合“点击化学”反应的RNA捕获策略能够不依赖RNA的poly(A)尾巴,更广泛的富集鉴定RP复合物。Huang等^[17]^开发的CARIC(click chemistry-assisted RNA-interactome capture)策略能富集全类型RP复合物,见图2。其主要思路是:首先将5-炔基尿苷(5-ethynyluridine, 5-EU,简称EU)与4SU代谢进同一条RNA中,EU提供了进行点击化学反应的炔基,然后在UV 365 nm照射下使RNA与RBP交联,接着利用叠氮与炔基的生物正交“点击化学”反应在EU的位置上引入生物素基团,最后利用生物素与链霉亲和素之间的强相互作用实现细胞中所有RP复合物的富集与捕获,其中一部分使用蛋白酶K处理进行RNA-seq分析,另一部分使用RNase A处理进行蛋白质组学分析。与之类似的,Bao等^[18]^开发的RICK(RNA interactome using click chemistry)策略也利用代谢标记结合“点击化学”反应将生物素标记在RNA上用于富集鉴定,不同之处是他们只将EU代谢进RNA,在254 nm UV条件下交联。利用这种基于“点击化学”的方法可以鉴定到除mRNA之外的各种类型的ncRNA,包括长非编码RNA(long non-coding RNA, lncRNA)、微小RNA(microRNA, miRNA)和核小RNA(small nuclear, snRNA),是方法学上的重大突破。然而,由于需要将非天然核苷代谢进RNA,这种策略面临和PAR-CLIP类似的局限性,例如仅限于细胞水平研究和存在一定的细胞毒性,并且分析的灵敏性很大程度上取决于EU的代谢标记效率。

**图2:**
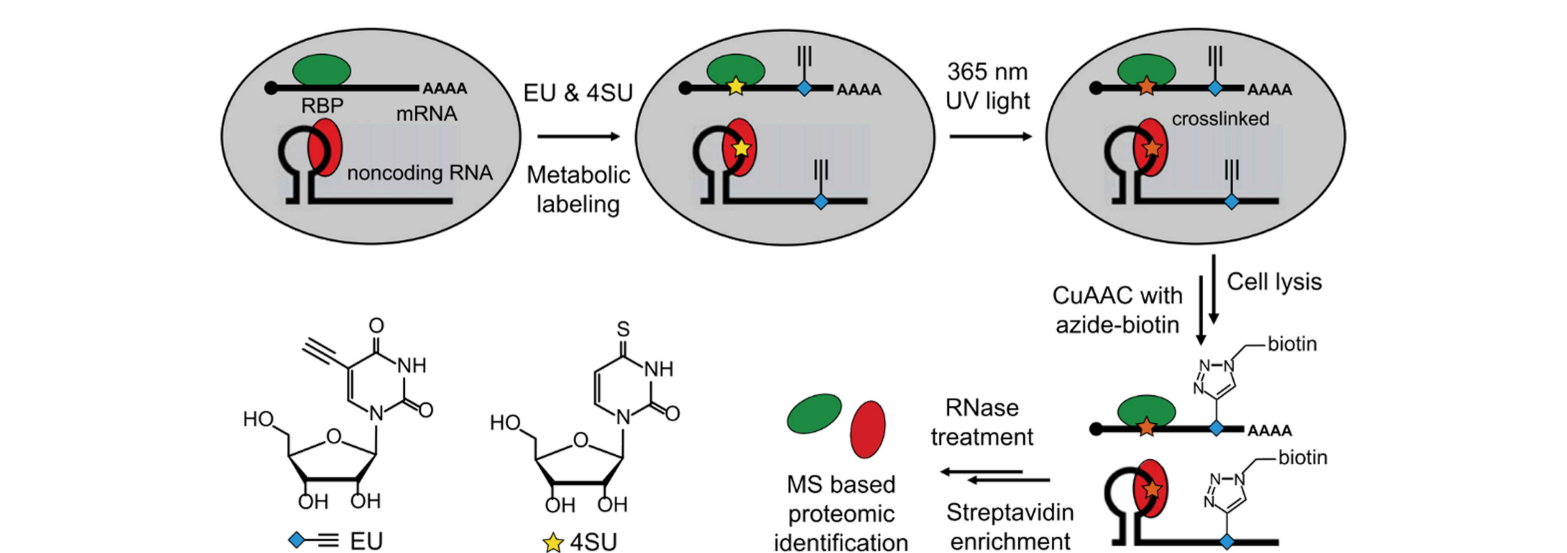
CARIC策略工作流程示意图^[17]^

### 1.3 基于相分离的富集策略

早期研究RNA提取时常采用基于酸性苯酚的相分离法^[19]^。首先破碎细胞,将核酸蛋白复合物中的蛋白质变性并释放出核酸,接着采用苯酚抽提,苯酚的诱导极化作用会使蛋白质内外翻转,疏水性侧链暴露在外部,极性残基翻转到内部,从而将水相中的蛋白质萃取出来。同时,由于DNA和RNA在特定pH值下的溶解度不同,低pH条件下(pH<5)的苯酚使RNA进入水相,而DNA维持不溶解的状态。最终在酸性苯酚萃取下RNA进入上层水相,而大多数DNA和蛋白质则保留在中间层或者下层有机相中。最近发展了一系列基于UV交联和相分离原理的富集策略,交联后的RP复合物会集中在水相与有机相之间的界面,再经过进一步纯化可以实现RP复合物的分离富集,见图3^[20]^。正交有机相分离(orthogonal organic phase separation, OOPS)策略^[21]^正是基于这种思路,使用酸性异硫氰酸胍-苯酚-氯仿(acidic guanidinium-thiocyanate-phenol-chloroform, AGPC)作为有机相,通过连续多次AGPC萃取后得到RP复合物,然后通过RNase消化RNAs获得分配到有机相的RBPs,最后通过质谱鉴定在HEK293、U2OS和MCF10A 3种人类细胞系中共鉴定到了1838个RBPs,包括926个推定的RBPs,其中约80%的RBPs与先前报道的不依赖poly(A)的策略(CARIC和RICK)结果一致,这说明OOPS具有更全面的分离富集RP复合体的能力,此外OOPS还可以进行RNA-蛋白质相互作用的动态分析。

**图3:**
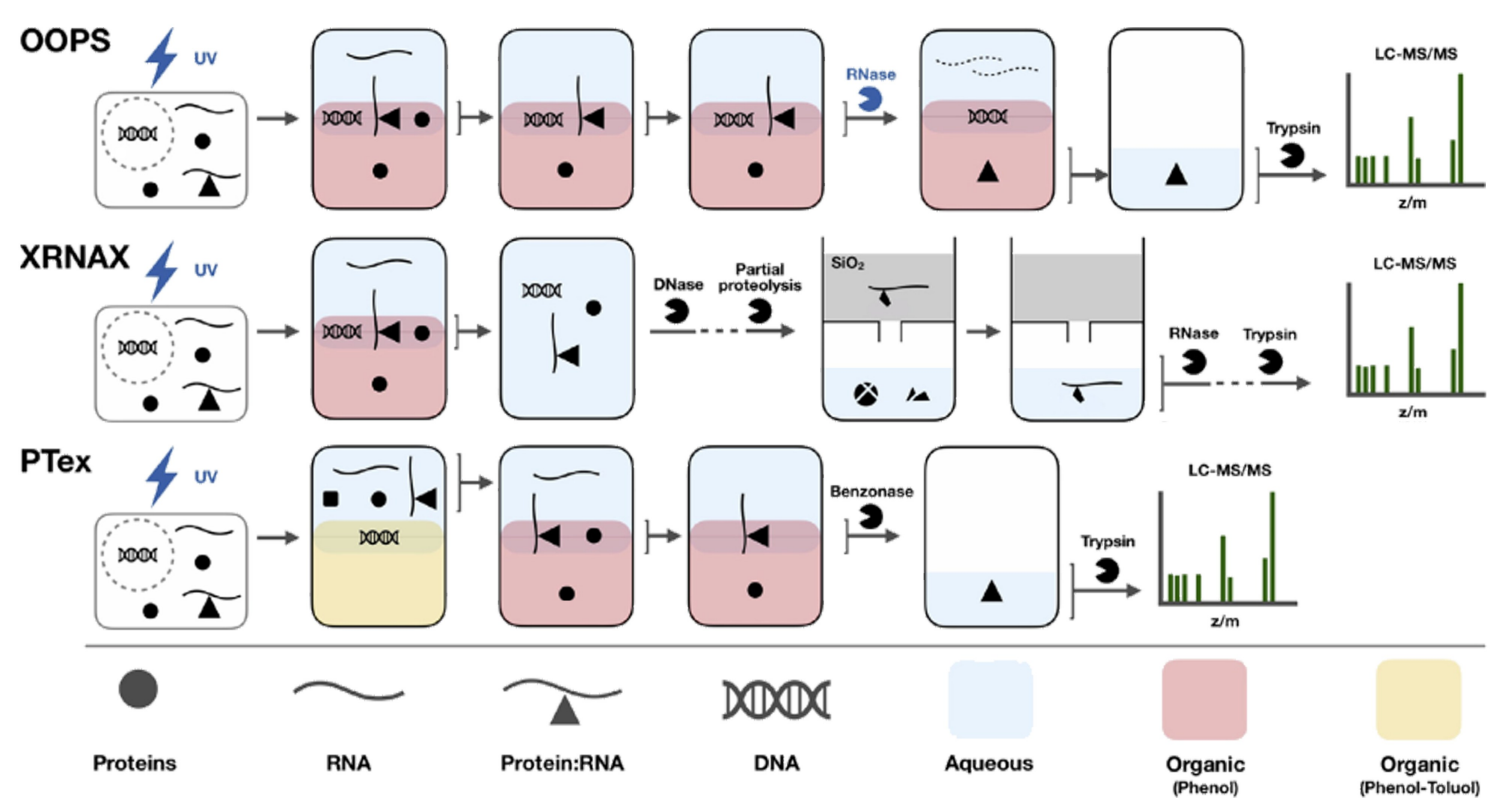
基于相分离的RP复合物富集方法^[20]^

另一种基于相分离的策略是苯酚-甲苯萃取(phenol-toluol extraction, PTex)策略^[10]^,不同之处是有机相为pH 7.0的苯酚-甲苯(50∶50, v/v)混合溶液。在这种体系下,RNAs、蛋白质和RP复合物分配在上层水相中,DNA和脂质在中间层,回收水相后与酸性苯酚混合进行多次萃取得到RP复合物。通过这种分离策略从HEK293细胞中鉴定出共3037个RBPs,回收率约为30%~50%。为了进一步提高RP复合物的富集选择性,一种新的策略XRNAX(protein-crosslinked RNA extraction)联合TRIzol(total RNA isolation)试剂相分离与二氧化硅实现RP复合物的富集^[22]^。TRIzol常用于总RNA分离纯化,能保持RNA的完整性,主要成分是苯酚。XRNAX的主要思路是:首先利用TRIzol将DNA、蛋白质和RP复合物分布在中间层,回收中间层后通过DNase消化DNA。由于硅胶柱在标准条件下可以保留RNA,但不保留与蛋白质交联的RNA,通过蛋白酶部分酶解得到RNA-肽段复合物使其可以保留在硅胶柱中,从而有效富集了RNA-肽段复合物。除去非交联的肽段后,对RBPs的富集选择性从69%增加到89%。结合在3种细胞系(MCF7、HeLa和HEK293)中的应用结果,共鉴定到1753个RBPs,其中有858个RBPs是3种细胞系共有的。

相分离策略不依赖于RNA特定序列,完全根据RP复合物的理化性质实现分离富集,然而由于利用了UV交联,相分离策略也面临着UV偏好性、穿透力弱、交联效率低等问题,而且糖蛋白具有与RP复合物类似的理化性质,可能会污染富集产物。

## 2 总结与展望

RP复合物富集策略的不断创新使方法学取得了重大进步,从而大大提高了RBP在不同物种中的覆盖深度,为基因表达和转录后调控的研究提供了重要的参考依据。本文对不同方法的优缺点进行了比较和讨论,以方便研究者们选择合适的策略来解决感兴趣的生物学问题。由于当前的RP复合物富集方法仍然存在效率低和操作繁琐等问题,因此迫切需要高效、易于实施并适用于不同类型样品的新方法。目前亟待解决的问题包括:1)基于UV或甲醛的交联策略仍存在选择性和交联效率有限等局限,因此需要开发新的交联剂或交联策略。2)目前已经成功鉴定出数千个RBPs,而与RNAs的结合RBP位点鉴定数量却较少(仅报道了几百个)。因此,需要更为特异的RNA-蛋白质交联策略和高灵敏度质谱分析方法。3)目前用于验证新发现的RBPs的方法通量较低,难以满足大规模验证RNA-蛋白质相互作用的重大需求。
